# Penile, scrotal, and gluteal vesicles

**DOI:** 10.1007/s10354-025-01100-z

**Published:** 2025-07-29

**Authors:** A. Kogler, K. Großschädl, B. Sadoghi

**Affiliations:** Department of Dermatology and Venereology, Medical University of Graz, Auenbruggerplatz 8, 8036 Graz, Austria

**Keywords:** Herpes zoster, Shingles, Herpes genitalis, Varicella zoster virus, Genital, Scrotal

## Abstract

**Background:**

Herpes zoster results from reactivation of the varicella zoster virus (VZV) and most frequently affects the thoracic dermatomes, especially T3 to T5.

**Case description:**

We present the case of a 61-year-old patient who presented with dysuria and grouped, partly crusted vesicles, some on an erythematous base on the glans penis, scrotum, and gluteal region. Differential diagnoses included herpes virus infection, bacterial balanoposthitis, syphilis, and contact dermatitis. A polymerase chain reaction (PCR) test for VZV was positive, confirming the diagnosis of herpes zoster.

**Conclusion:**

Sacral herpes zoster, including dermatomes such as S2, S3, and S4, is very rarely reported. This case highlights the importance of atypical presentations of herpes zoster and emphasizes the diagnostic and therapeutic considerations relevant for clinicians managing them.

## Case presentation

A 61-year-old male patient experienced dysuria, followed by the development of a solitary erythematous macule in the gluteal area the very next day. He presented to his general practitioner, where systemic antibiotic treatment with amoxicillin/clavulanic acid (875/125 mg twice daily) was initiated 2 days before presentation to our department. However, the clinical symptoms worsened. Two days after starting the antibiotic treatment, the patient developed pruritic, burning, and painful vesicles and was referred to our Department of Dermatology.

The sexual history revealed that his last sexual contact had occurred several years ago. He identified as heterosexual and did not engage in chemsex. He denied any trauma or exposure to irritating substances. His past medical history included Crohn’s disease, hypothyroidism, chronic nicotine use, and polyneuropathy. His long-term medications included pantoprazole, pregabalin, levothyroxine sodium, and cholecalciferol D3.

**Fig. 1:**
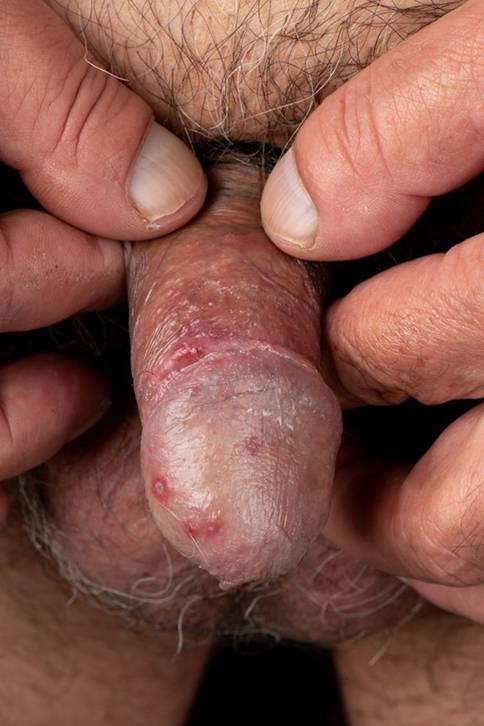
Grouped, partly erosive vesicles on an erythematous base on the glans penis

**Fig. 2:**
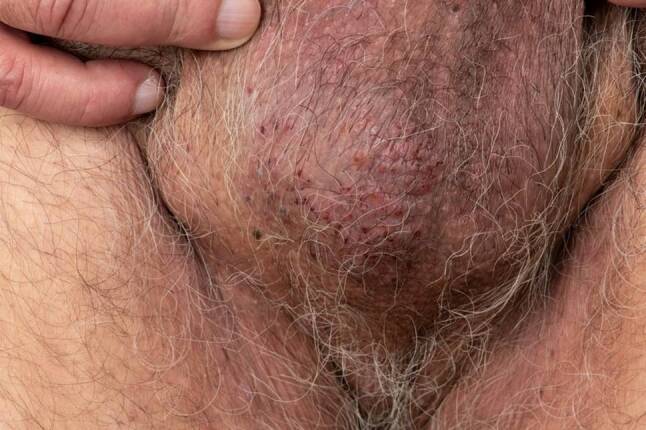
Grouped, partly crusted vesicles on an erythematous base on the scrotum

**Fig. 3:**
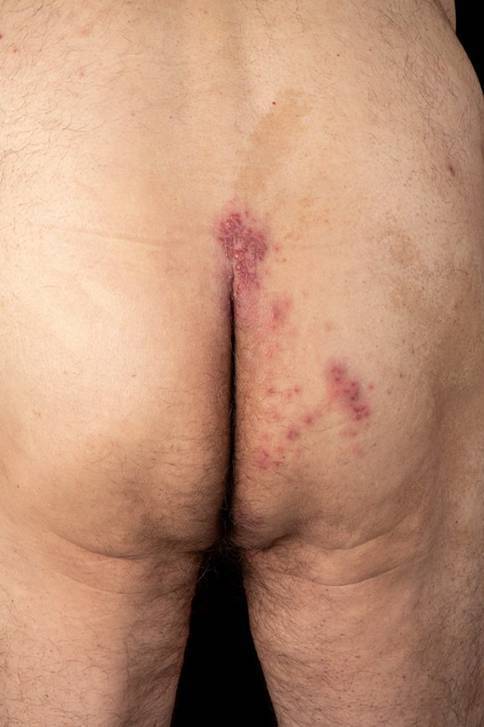
Grouped vesicles on an erythematous base in the gluteal area

On clinical examination, grouped vesicles on an erythematous base, some with crusting, were observed on the glans penis (Fig. 1) and scrotum (Fig. 2). A more detailed examination revealed vesicles in the gluteal area (Fig. 3). Additionally, scattered solitary vesicles were noted on the trunk, upper back, and right thigh.

Laboratory tests revealed an elevated C‑reactive protein (CRP) level of 25.7 mg/L (reference range < 5 mg/L), while other laboratory values, including the differential blood count, were within normal limits. Urinalysis demonstrated mild microhematuria (erythrocytes +/+). Urine culture (uricult) revealed no evidence of a urinary tract infection (UTI). Human immunodeficiency virus (HIV) testing was not performed at the time of presentation, as there were no clinical or other indicators in the medical history. A subsequent HIV test performed 3 months later was negative.

The clinical picture was highly suspicious for varicella zoster virus infection (S3 dermatome) and systemic antiviral treatment was immediately started with acyclovir at 5 mg/kg body weight three times daily for a duration of 10 days. Locally, a single daily application of a suspension containing talc and zinc oxide was used on the gluteal and aberrant vesicles, and a cream containing dexpanthenol and chlorhexidine dihydrochloride was applied to the glans penis. The polymerase chain reaction (PCR) test for varicella zoster virus (VZV) was positive, confirming the suspected diagnosis of herpes zoster.

For herpetic neuralgia, pain therapy was initiated, consisting of metamizole 500 mg on an as-needed basis, up to three times daily; pregabalin in an ascending dosage regimen starting at 100 mg twice daily; and sustained-release hydromorphone at 2 mg twice daily.

For further examination, a hemoccult test, chest X‑ray, prostate-specific antigen (PSA), and upper abdominal ultrasound were performed and did not show any abnormalities.

Around day four of illness, the patient reported abdominal discomfort. Subsequent colonoscopy performed approximately 10 days after the onset of herpes zoster revealed severe inflammatory activity with stenosis in the neoterminal ileum (Rutgeerts i4). Magnetic resonance enterography showed a short stenosis in the neoterminal ileum and a 6.6 cm inflamed, stenotic segment with skip lesions in the mid-abdomen, consistent with active Crohn’s disease.

## Discussion

### Etiology and pathogenesis

Herpes zoster results from reactivation of the varicella zoster virus (VZV), also referred to as human herpesvirus 3 (HHV-3) [1]. Varicella zoster virus is a member of the *Herpesviridae* family and is classified as an alphaherpesvirus, closely related to herpes simplex viruses (HSV‑1 and HSV-2) [1]. Following initial infection, which presents clinically as varicella (chickenpox), the virus disseminates from mucocutaneous tissues to neurons within cranial nerve ganglia, dorsal root ganglia, enteric ganglia, and autonomic ganglia, where it persists in a latent state until reactivation [2]. Upon reactivation, the dormant VZV spreads from sensory ganglia along the sensory nerves, resulting in the development of herpes zoster in the corresponding dermatome [2, 3].

Reactivation of varicella zoster virus may occur spontaneously or be triggered by stress, fever, radiation, trauma, or a weakened immune system [4]. In over 90% of cases, initial symptoms (prodrome) include itching, tingling, tenderness, hyperesthesia, or intense pain [4]. In the present case, the patient experienced a severe flare of Crohn’s disease at the time of viral reactivation, as evidenced by colonoscopy and magnetic resonance enterography revealing significant inflammatory activity and stenosis in the neoterminal ileum. The patient had a known history of Crohn’s disease treated intermittently with azathioprine over the previous 2 years; however, the medication had been discontinued approximately 2 weeks prior to the onset of herpes zoster due to perceived intolerance. Thus, at the time of VZV reactivation, the patient was not under immunosuppressive therapy. This suggests that the systemic inflammatory activity associated with the Crohn’s disease flare itself may have acted as an immunological stressor, contributing to VZV reactivation even in the absence of active immunosuppression.

### Epidemiology

Epidemiological studies show that the prevalence of herpes zoster increases markedly after the age of 50, with approximately 50% of individuals reaching 85 years experiencing at least one episode during their lifetime [5].

Recent epidemiological data from Germany demonstrate a significant increase in the incidence of herpes zoster over the past decade, especially among older adults.

Lebedeva and Dissemond (2022) analyzed inpatient cases from 2009 to 2022 and reported a notable rise in hospital admissions due to herpes zoster. The study highlights that demographic aging and declining cellular immunity contribute to this trend [6].

### Dermatomal distribution of herpes zoster

The sacral dermatomes (S2–S4) are involved in only around 5% of herpes zoster cases, whereas thoracic dermatomes are affected in approximately 56%, cervical in 17%, trigeminal in 12%, and lumbar in 10% of cases, according to epidemiological studies [7]. The reason for this low frequency is currently unknown. Although some experimental and clinical studies have explored general mechanisms of VZV latency and reactivation—such as differences in immune surveillance, glial cell function, and neuronal subtypes in dorsal root ganglia—these aspects have not been examined in relation to specific spinal segments [8, 9].

### Diagnostic approach

Many varicella zoster virus infections can be diagnosed clinically. However, atypical rashes may require direct immunofluorescence for VZV antigen or PCR for VZV DNA from lesion samples. According to current guidelines, routine HIV testing is recommended for individuals under the age of 50 [10].

### Complications

Herpes zoster can result in persistent pain (postherpetic neuralgia, PHN) and may cause serious neurological, ocular, and systemic complications. These include meningoencephalitis, myelitis, cranial nerve palsies, vasculopathy, keratitis, and retinopathy as well as visceral involvement such as gastrointestinal ulcers, hepatitis, and pancreatitis [1, 2, 11]. Involvement of the sacral dorsal root ganglia, as in the present case, can result in autonomic dysfunction, presenting with urinary retention, erectile dysfunction, or loss of anal and bulbocavernosus reflexes [12].

The risk of developing PHN increases with age and the severity of the acute symptoms [13, 14]. It is also elevated in individuals with chronic conditions such as diabetes or respiratory disease and possibly in immunocompromised patients, although the evidence in the latter group remains inconsistent [13, 14].

The incidence of PHN appears largely independent of the affected dermatome. Hope-Simpson observed that PHN occurs with a similar frequency across different dermatomal regions, including sacral involvement, but may be of shorter duration when the lumbosacral area is affected [5].

### Therapeutic management

It is recommended that patients with zoster receive systemic antiviral treatment, particularly those who are immunocompromised, aged 50 years or older, have lesions on the face or eyes, experience severe rashes, or have other zoster-related complications [1, 3, 15]. The standard antiviral treatment (e.g., acyclovir, valacyclovir) and pain management approaches apply similarly regardless of the dermatome.

### Prophylaxis

In Austria, vaccination against herpes zoster is recommended for individuals aged 50 and older, as outlined in the national immunization plan. In addition, individuals from 18 years of age with an increased risk—such as those with weakened immune systems—are also encouraged to get vaccinated. This helps reduce the risk of developing shingles and complications thereof, such as postherpetic neuralgia. The vaccine is administered as a single-dose shot [16]. In our case report, the patient’s varicella zoster virus vaccination status was not documented in the available medical records.

Current guidelines recommend that vaccination against varicella zoster virus should be offered not only to individuals without prior herpes zoster but also to patients who have already experienced and recovered from an episode of herpes zoster. This is because natural infection does not confer lifelong immunity, and vaccination has been shown to reduce the risk of recurrence significantly. According to recent expert recommendations, vaccination should be administered once the acute infection has fully resolved [17]. Two vaccines have been developed for the prevention of herpes zoster: a live attenuated vaccine (Zostavax®), administered as a single dose, and an adjuvanted, recombinant inactivated vaccine (Shingrix®), given in a two-dose schedule. Due to its higher efficacy and longer-lasting protection, the inactivated vaccine is the preferred option recommended by health authorities [16, 18, 19].
